# Response to Commentary: The framework for systematic reviews on psychological risk factors for persistent somatic symptoms and related syndromes and disorders (PSY-PSS)

**DOI:** 10.3389/fpsyt.2024.1359434

**Published:** 2024-02-08

**Authors:** Paul Hüsing, Abigail Smakowski, Bernd Löwe, Maria Kleinstäuber, Anne Toussaint, Meike C. Shedden-Mora

**Affiliations:** ^1^ Department of Psychosomatic Medicine and Psychotherapy, University Medical Center Hamburg-Eppendorf, Hamburg, Germany; ^2^ Department of Psychology, Utah State University, Logan, UT, United States; ^3^ Department of Psychology, Medical School Hamburg, Hamburg, Germany

**Keywords:** somatic symptom and related disorders, systematic reviews, somatoform disorders, bodily distress disorder, psychological factors, persistent somatic symptoms, medically unexplained symptoms

## Introduction

Persistent somatic symptoms (PSS) are common in all fields of medicine. Current classification systems for mental disorders in this field, i.e. Somatic Symptom Disorder (SSD; DSM-5) or Bodily Distress Disorder (BDD; ICD-11), now stress the relevance of psychological features associated with the physical complaints. It is well known that psychological criteria are among the relevant risk factors for the development and/or worsening of persistent physical symptoms, however, the selected diagnostic criteria remain subject to debate. Numerous psychological concepts have been studied and discussed in the scientific field. However, empirical evidence remains scattered, individual factors have not been reviewed systematically, and longitudinal studies to allow for causal inference are scarce. In our framework for systematic reviews on psychological risk factors for persistent somatic symptoms and related syndromes and disorders (PSY-PSS) (1), we summarized current knowledge regarding psychological variables relevant to the development and maintenance of persistent somatic symptoms (PSS). The framework provides two lists, one of them with 83 relevant symptoms, syndromes and disorders (list 1) and the other with 120 psychological factors, categorized into 42 subcategories and 7 main categories (list 2). Further, we invited other researchers working in the field of PSS to use and also improve our lists by adding terms and constructs which we might have missed in our initial search. Following up on our invitation, Berens and colleagues (2) rightly pointed out that so far, our list of psychological variables did not contain any factors related to personality functioning and mentalizing, although there is scientific evidence for these concepts from the field of PSS, which the authors convincingly explain in their commentary. By adding these important psychodynamic concepts, the authors helped to improve the PSY-PSS framework.

## The new terms

**Figure 1:**
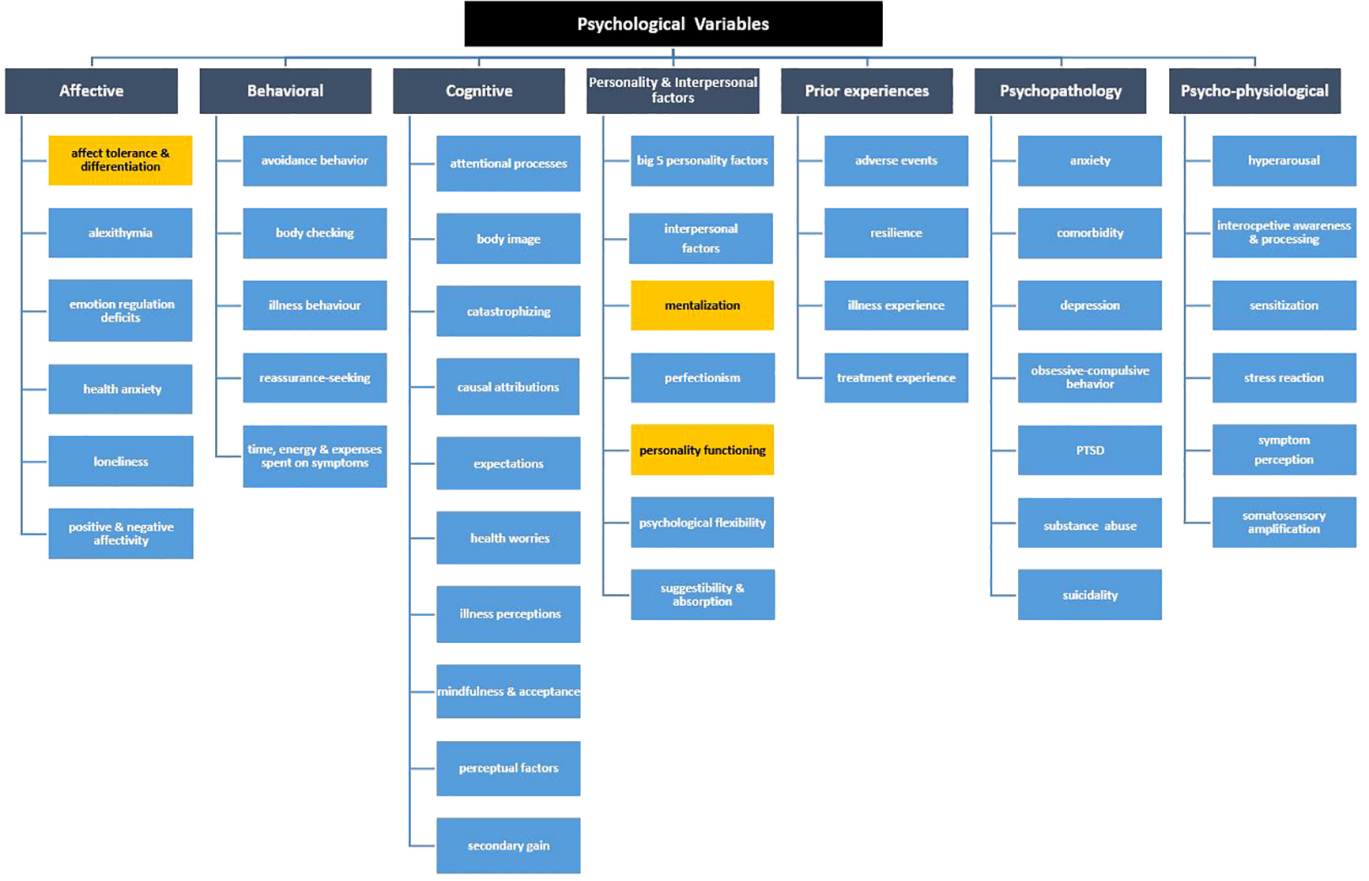
Updated tree diagram with psychological variable main categories and subcategories, changes highlighted in yellow (3).

In their commentary, the authors argue that mentalization, reflective functioning, embodied mentalization, affective theory of mind, psychic structure, personality functioning, affect tolerance and affect differentiation should be added to the list of psychological search terms with potential relevance to diagnosis and treatment of PSS. They provide empirical evidence and stress the importance of these specific concepts with regard to the diagnostic classifications of troublesome and persistent somatic symptoms, i.e., somatic symptom disorder by DSM-5 and bodily distress disorder by ICD-11. We highly appreciate the input by Berens and colleagues and agree to include the proposed terms in the list of psychological variables relevant to the development and maintenance of PSS and associated clinical outcomes such as patient impairment or health care utilization. In collaboration with the Berens and colleagues, we added the respective terms to our tree diagram in the subcategories of “affective factors”, i.e., “affect intolerance & differentiation”, and “personality and interpersonal factors”, i.e., “mentalization” and “personality function” (**Figure 1**).

## Use of the PSY-PSS and future directions

The lists of relevant symptoms, syndromes and disorders, and psychological factors, respectively, can be found on the Open Science Framework (OSF) and will be updated regularly based on incoming evidence and evolvement of the research field (3). Interested researchers may use the PSY-PSS lists and search terms and contact us if they feel that any syndromes or psychological factors of relevance are still missing from our lists. PSY-PSS is an open framework that can be complemented by further promising psychological risk factors. It is the basic idea of the framework to enable researchers to generate systematic evidence on a variety of potentially relevant psychological risk factors, whereas the lists do not in themselves constitute proof of the relevance of the psychological variables listed. Given the dynamic nature of the field and the still existing gasps in knowledge, we hope that our contribution might enable interested researchers to conduct their work with less expense, thus generating more empirical data and insights on persistent somatic symptoms, their aetiology and consequences for patients.

## Discussion

In their commentary, Berens et al. pointed out several important psychological variables mostly derived from psychodynamic research that were missing in our proposed PSY-PSS framework. We agree on their importance for future studies in the research field and integrated them into our updated lists. It is our aim to provide a comprehensive list of all potentially relevant psychological terms associated to PSS. Most of them need further studying as the empirical evidence is scarce. Recent debates on the role of psychological factors within medical conditions such as long-COVID or CFS underline the need for high quality research in this field. A uniform language and conceptualisation provided by our search term lists is an important aid to conduct comparable studies and synthesize existing data. We hope that the updated PSY-PSS framework will facilitate the work of research groups interested in PSS and related syndromes and disorders, and that future generations of patients will benefit from the insights derived from it.

## Author contributions

PH: Writing – original draft, Writing – review & editing. AS: Writing – review & editing. BL: Writing – review & editing. MK: Writing – review & editing. AT: Writing – original draft, Writing – review & editing. MS-M: Writing – original draft, Writing – review & editing.
